# LIMD1 Increases the Sensitivity of Lung Adenocarcinoma Cells to Cisplatin via the GADD45α/p38 MAPK Signaling Pathway

**DOI:** 10.3389/fonc.2020.00969

**Published:** 2020-07-10

**Authors:** Xiaofei Zeng, Hong Wang, Dongsheng He, Weikun Jia, Ruidong Ma

**Affiliations:** Department of Cardiothoracic Surgery, First Affiliated Hospital of Chengdu Medical College, Chengdu, China

**Keywords:** LIMD1, p38 MAPK, lung adenocarcinoma, cisplatin, apoptosis

## Abstract

**Objective:** To investigate the effect of LIM domain-containing protein 1 (LIMD1) on the sensitivity of lung adenocarcinoma cells to cisplatin and explore the mechanism.

**Methods:** A549 and H1299 cells were transfected with lentivirus to establish LIMD1-overexpressing cell lines and their respective controls. The protein expression of DNA damage-inducible 45 alpha (GADD45α) and p38 mitogen-activated protein kinase (MAPK) was detected by Western blot. The survival of A549-vec, A549-LIMD1, H1299-vec, and H1299-LIMD1 cells after cisplatin treatment was observed by CCK-8, and the viability was calculated accordingly. Then, SB203580 was used to inhibit the activity of the p38 MAPK signaling pathway, after which the survival of A549-vec, A549-LIMD1, H1299-vec, and H1299-LIMD1 cells in response to cisplatin was observed again by CCK-8, and the viability was calculated accordingly.

**Results:** When LIMD1 was overexpressed in A549 and H1299 cells, the levels of GADD45α and p-p38 MAPK were increased, but total p38 MAPK expression showed no significant change. After adding 30 μM cisplatin, the optical density (OD) values of A549-LIMD1 and H1299-LIMD1 cells were significantly lower than those of their respective controls at 24, 48, and 72 h. The viability of A549-LIMD1 and H1299-LIMD1 cells was significantly lower than that of their respective controls at all the times tested (*p* < 0.05). The Western blot results showed that the expression of apoptotic proteins cleaved caspase 3 and cleaved PARP in cisplatin-treated A549-LIDM1 and H1299-LIMD1 cells was significantly higher than that in their respective control cells. Flow cytometry showed that the apoptosis rates of A549-LIMD1 and H1299-LIMD1 cells were significantly higher than those of their respective controls (*p* < 0.05). SB203580 significantly inhibited the activation of the p38 MAPK signaling pathway in lung adenocarcinoma cells; however, neither the OD values nor the viability of A549-LIMD1 cells and H1299-LIMD1 cells showed no significant difference from those of their controls at 24, 48, and 72 h after cisplatin and SB203580 treatment (*p* > 0.05 for both). Western blot analysis showed that after SB203580 was added, the expression of cleaved caspase 3 and cleaved PARP in A549-LIMD1 and H1299-LIMD1 cells presented no significant difference compared with that in their respective controls.

**Conclusion:** LIMD1 increases the sensitivity of lung adenocarcinoma cells to cisplatin by activating the GADD45α/p38 MAPK signaling pathway.

## Introduction

Lung adenocarcinoma is a common malignant tumor in China, with the characteristics of occult onset and rapid progression. Currently, there are no effective treatments, which results in patients having a low survival rate (1). Lung adenocarcinoma is less sensitive to common chemotherapeutic drugs, resulting in poor outcomes, but the mechanism of this resistance is not yet clear (2). Therefore, it is of clinical significance to explore the mechanism of resistance and find effective targets to increase the sensitivity of lung adenocarcinoma cells to chemotherapeutic drugs.

P38 MAPK is a mitogen-activated protein kinase (MAPK) (3), and its signaling pathway in mammals is involved in many important intracellular activities. Activation of the p38 MAPK signaling pathway enables cells to transduce multiple extracellular signals and thus generate a variety of biological responses, such as inflammation, differentiation, cell proliferation, and apoptosis, depending on the types of tissues and signals (4, 5). Studies have revealed that the p38 MAPK signaling pathway plays a critical role in regulating the sensitivity of tumor cells to drugs, e.g., activation of the p38 MAPK signaling pathway in triple-negative breast cancer cells increases the sensitivity of tumor cells to doxorubicin and inhibits tumor migration and invasion (6). Moreover, persistent activation of the JNK/p38 MAPK signaling pathway induced by cisplatin leads to apoptosis of ovarian cancer cells (7). Inhibiting the JNK/p38 MAPK signaling pathway can attenuate cisplatin-induced apoptosis of ovarian cancer cells, suggesting that the JNK/p38 MAPK signaling pathway is involved in the response of tumor cells to cisplatin (8). P38 MAPK signaling pathway activity is controlled by a regulatory network. The growth arrest and DNA damage-inducible 45 alpha (GADD45α) protein is an important molecule upstream of the p38 MAPK signaling pathway (9–11), and the level of p-p38 MAPK is decreased upon interference with GADD45α expression, indicating that GADD45α can activate the p38 MAPK signaling pathway (12, 13). However, the role of the p38 MAPK signaling pathway in regulating the sensitivity of tumors to chemotherapeutic drugs and the mechanisms involved remain unclear.

LIM domain-containing protein 1 (LIMD1), a tumor suppressor gene encoded at chromosome 3p21.3, is often deleted in many solid malignant tumors (14). LIMD1 is a member of the Ajuba family of proteins, all of which contain the LIM domain (15). Studies have reported that LIMD1 expression is low in human lung cancer cells due to genetic or epigenetic modifications (16, 17). Sharp et al. confirmed that in cell and animal experiments, LIMD1 overexpression in tumor cells can inhibit the growth of tumor cells; thus, LIMD1 is regarded as a tumor suppressor gene (16, 18). Sharp et al. also found that as a target gene of HIF-1, LIMD1 participates in the degradation of HIF-α under hypoxic conditions through a negative feedback loop, thereby exerting the anticancer effect (19). In addition, LIMD1 interacts with the Rb protein to inhibit E2F1-mediated transcription through synergy (18). Spendlove et al. found that the content of LIMD1 in the nucleus is related to the prognosis of breast cancer patients; the lower the content, the worse the prognosis (17). Ghosh et al. reported that LIMD1 gene mutations are an independent risk factor for the prognosis of patients with head and neck squamous cell carcinoma, and patients with a LIMD1 mutation usually have a poor prognosis (20). The aforementioned studies have shown that LIMD1 plays a critical regulatory role in tumor progression, and the mechanism of LIMD1 is studied; however, whether LIMD1 expression level is correlated with chemotherapy sensitivity is not investigated. This study investigated the role of LIMD1 in regulating the sensitivity of lung adenocarcinoma cells to cisplatin, and we also explored its mechanism of enhancing sensitivity.

## Materials and Methods

### Cells and Reagents

The lung adenocarcinoma cell lines A549 and H1299 were purchased from the Cell Bank, Chinese Academy of Sciences (Shanghai). RPMI and DMEM were purchased from HyClone Co., USA. Fetal bovine serum was purchased from Yikesai Biotechnology Co., Ltd., Taicang, China. Primary antibodies targeting LIMD1, GADD45α, p38 MAPK, p-p38 MAPK, cleaved caspase 3, cleaved PARP, and GAPDH as well as anti-rabbit secondary antibody were purchased from Cell Signaling Technology Co., USA. The CCK-8 kit and SDS-PAGE gel preparation kit were purchased from Beyotime Biotechnology Co., China. The p38 MAPK inhibitor SB203580 was purchased from MedChemExpress Co., USA. Puromycin was purchased from Beyotime Biotechnology Co., China. An apoptosis detection kit was purchased from Jiangsu Kaiji Biotechnology Co., Ltd., China. The microplate reader was purchased from Thermo Scientific (USA), whereas the flow cytometer was obtained from Beckman Coulter Co., Ltd. (USA). The lentiviral packaging system, including overexpressed lentiviral vector plasmid pLVX-IRES-puro, helper plasmids pSPAX2, and pMD2.G, was purchased from the Chengdu Transvector Biotechnology Company (China). The transfection reagent Lipofectamine 3000 was purchased from Thermo Fisher Scientific (USA), and the plasmid extraction kit was purchased from OMEGA (USA). The LB liquid medium powder was purchased from Sangon Biotech Co., Ltd., Shanghai, China, and Polybrene was purchased from Beijing Solarbio Science & Technology Co., Ltd., China.

### Packaging of Lentivirus

The PubMed database was searched for the gene sequence of LIMD1, and its CDS was identified for gene synthesis. After the gene products were subjected to restriction enzyme digestion and ligation, the fragments were cloned into the pLVX-IRES-puro lentiviral vector, with empty vector serving as a control for the overexpression group. The 293T cells were transfected with lentivirus (viral vector:pMD2.G:pSPAX2, 4:1:3). The medium was changed at 6 h after transfection, and virus particles were collected at 48–72 h after transfection, centrifuged at 3,500× *g*, and stored at |80°C until further use.

### Lentivirus Infection

Cells were passaged and cultured into six-well plates. The lentivirus was thawed at room temperature and centrifuged at 4,000× *g* for 10 min at 4°C, and the supernatant was transferred to a fresh tube and mixed with polybrene at a final concentration of 8 μg/ml. The original medium in the six-well plate was discarded; 3 ml of lentivirus supernatant was added to each well and placed in an incubator at 37°C for 1 h. Then, the lentivirus supernatant was discarded, and the infection was repeated once. After the viral medium was removed, RPMI medium was added. Three to four days later, RPMI medium containing 2 μg/ml of puromycin was added for screening.

### Cell Transfection

siRNA targeting p38 MAPK and its scramble control were purchased from the Shanghai Genepharma Company. The transfection was performed as previously described (21). Cells were passaged at a density of 70% and transfected with Lipofectamine 3000 reagent (Life Technologies) on the following day according to the manufacturer's instructions.

### Western Blot Analysis to Determine Protein Expression

After the protein concentrations in the samples were measured, 30 μg of protein per sample was aliquoted. Then, loading buffer was added to the samples and mixed before the tube was placed in a dry block heater at 100°C for 7 min. Then, the samples were loaded onto an SDS-PAGE gel and electrophoresed at 100 V for 100 min. Proteins were subjected to semidry transfer to a PVDF membrane at 10 V for 90 min, after which 5% skim milk powder in TBST solution was added to the membrane and incubated at room temperature for 1 h. The skim milk solution was used to dilute the primary antibodies, and then the bands were excised from the membrane, wrapped with transparent plastic, treated with primary antibody, and incubated at 4°C overnight. Any residual primary antibody was recovered the next day. The membranes were washed four times with TBST solution (5 min per wash). The secondary antibody was prepared with skim milk powder and incubated with the membranes for 2 h at room temperature, after which they were washed four times with TBST solution (5 min per wash). The protein bands were exposed after treatment with the developer solution.

### CCK-8 Kit to Measure Cell Viability

Cells were digested, resuspended, counted, passaged, and seeded into 96-well plates at a density of 10,000 cells per well. CCK-8 solution was added at a volume ratio of 1:10 in each well, and the plates were incubated for 2 h. The optical density (OD) was measured at a wavelength of 450 nm. The cells were seeded in a 96-well plate on the first day, the plate was placed in the incubator for the next day, and CCK-8 detection was performed on the third day. The value (at 0 h) was set as the reference value, at which point cisplatin was added to a final concentration of 30 μM. The CCK-8 detection was repeated at 24, 48, and 72 h after cisplatin treatment. The cell growth curve was drawn and calculated as follows: Cell survival (%) = [A (dosage) – A (blank)]/[A (0 dosage) – A (blank)] ×100%,

where A (dosage) is the OD of the wells containing cells, CCK solution, and drug,

A (blank) is the OD of the wells containing the medium and CCK solution only, and

A (no dosage) is the OD of the wells containing cells and CCK solution only.

### Flow Cytometry to Detect Apoptosis

Cells were digested with EDTA-free trypsin, resuspended and centrifuged, and the supernatant was discarded. Then, after the cells were washed one to two times with PBS solution and centrifuged, the supernatant was discarded, and the cell pellet was stained according to the standard protocols, incubated away from light for 15 min and assessed by flow cytometry.

### Statistical Analysis

Data processing was performed using SPSS 18.0 statistical software. Data are expressed as the mean ± SD. The results of the CCK-8 test were analyzed using a *t*-test.

## Results

### Overexpressing LIMD1 Significantly Increased Lung Cancer Cells' Sensitivity to Cisplatin

**Figure 1:**
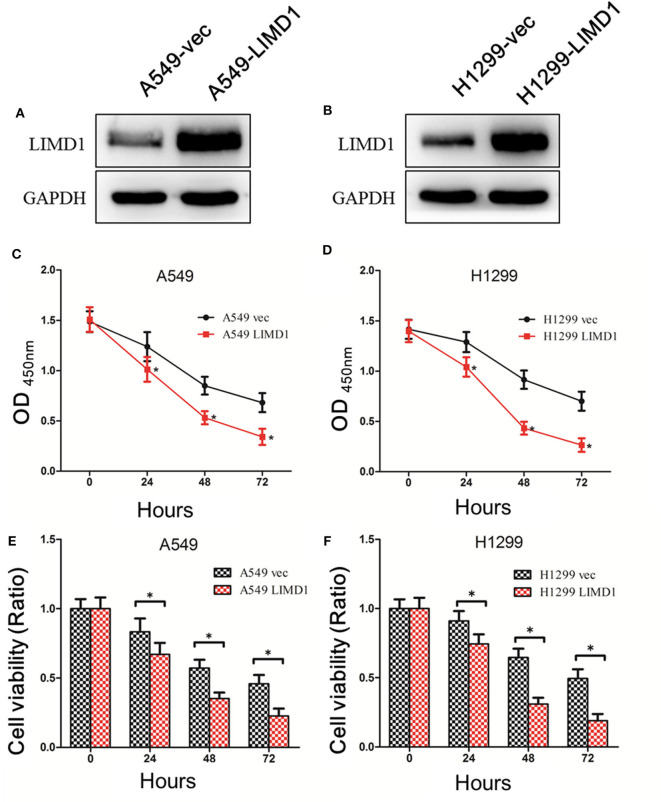
Overexpressing LIMD1 significantly increased lung cancer cells' sensitivity to cisplatin. **(A)** LIM domain-containing protein 1 (LIMD1) expression in A549 cells with LIMD1 overexpression and in control cells transduced with empty vector as detected by Western blot. **(B)** LIMD1 expression in H1299 cells with LIMD1 overexpression and in control cells transduced with empty vector as detected by Western blot. **(C)** Cell growth after cisplatin treatment detected by CCK-8. Optical density (OD) values are measured 24, 48, and 72 h after drug administration, and the data were statistically analyzed. **(D)** After the H1299-vec and H1299-LIMD1 cells were treated with 30-μM cisplatin, the OD values were measured before and 24, 48, and 72 h after drug administration, and the data were statistically analyzed. **(E)** The relative viability of A549-vec and A549-LIMD1 cells was calculated according to the OD values and was statistically analyzed. **(F)** The relative viability of H1299-vec and H1299-LIMD1 cells were calculated according to the OD values and were statistically analyzed. *A statistically significant difference at *P* < 0.05.

By performing lentivirus infection and screening, we successfully constructed cell lines overexpressing LIMD1 (A549-LIMD1, H1299-LIMD1) and their respective controls (A549-vec and H1299-vec). Western blotting was subsequently used to verify the overexpression and showed that A549-LIMD1 and H1299-LIMD1 cells had significantly higher levels of LIMD1 than did their respective control cells (Figures 1A,B).

First, 30-μM cisplatin was administered to A549-vec, A549-LIMD1, H1299-vec, and H1299-LIMD1 cells, and cell viability was detected by CCK-8 before treatment and at 24, 48, and 72 h after treatment. The viability of cells was calculated accordingly.

As shown in Figure 1C, the OD values were significantly different between the A549-LIMD1 and A549-vec cells after cisplatin treatment (*p* < 0.05), with the A549-LIMD1 cells presenting significantly lower values than the A549-vec cells at 24, 48, and 72 h after cisplatin treatment. The viability of A549-vec and A549-LIMD1 cells was calculated based on the OD values and presented a significant difference at 24, 48, and 72 h after cisplatin treatment (*p* < 0.05) (Figure 1E), with the A549-LIMD1 cells having significantly lower values than A549-vec cells at all three time points.

As shown in Figure 1D, the OD values were significantly different between the H1299-LIMD1 and H1299-vec cells after cisplatin treatment (*p* < 0.05), with the H1299-LIMD1cells presenting significantly lower values than the H1299-vec cells at 24, 48, and 72 h after cisplatin treatment. The viability of H1299-vec and H1299-LIMD1 cells were calculated based on the OD values and presented significant differences at 24, 48, and 72 h after cisplatin treatment (*p* < 0.05) (Figure 1F), with the H1299-LIMD1 cells having significantly lower values than H1299-vec cells at all three time points.

The aforementioned results showed that the sensitivity of A549 and H1299 cells to cisplatin was significantly increased after overexpression of LIMD1.

**Figure 2:**
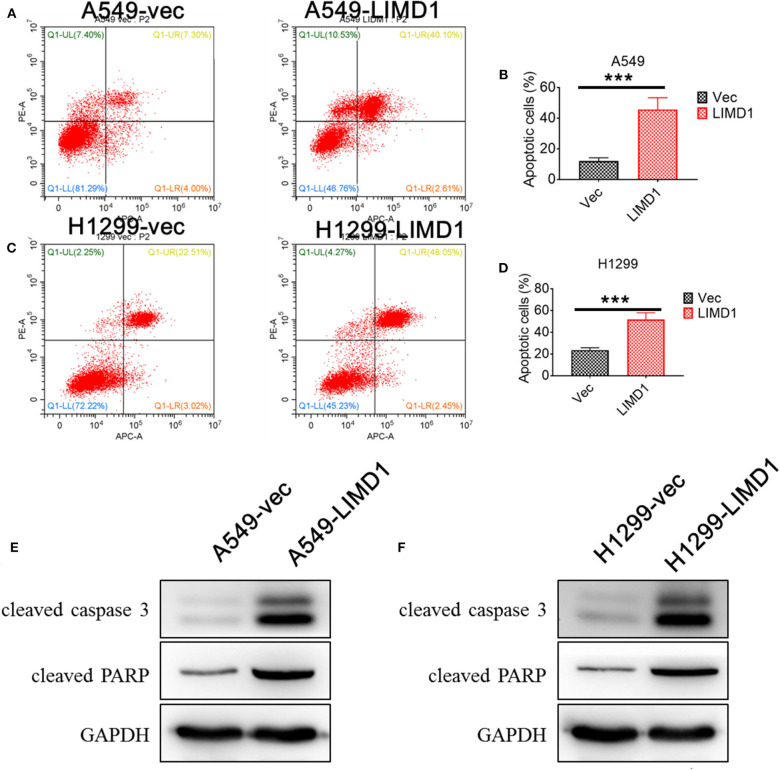
LIMD1 overexpression promoted cell apoptosis. Apoptosis levels of A549-vec and A549-LIMD1 **(A,B)**, H1299-vec and H1299-LIMD1 cells **(C,D)** after cisplatin treatment were determined by flow cytometry. The expression of proapoptotic proteins in A549-vec and A549-LIMD1 cells **(E)** and in H1299-vec and H1299-LIMD1 cells **(F)** was detected by Western blot. ***A statistically significant difference at *P* < 0.001.

Subsequently, flow cytometry and Western blotting were performed to detect the apoptosis levels of A549-vec, A549-LIMD1, H1299-vec, and H1299-LIMD1 cells in response to cisplatin treatment. The cells showed apoptosis at varying degrees after cisplatin treatment (Figure 2), with the apoptosis rate in the A549-LIMD1 group (42.71%) being significantly higher than that in the A549-vec group (11.30%) and the proportion of normal cells in the A549-LIMD1 group (46.76%) being significantly lower than that in the A549-vec group (81.29%). Similarly, the apoptosis rate in the H1299-LIMD1 group (50.5%) was significantly higher than that in the H1299-vec group (25.53%), while the proportion of normal cells in the H1299-LIMD1 group (45.23%) was significantly lower than that in the H1299-vec group (72.22%). The results of the Western blot analysis revealed that the expression levels of cleaved caspase 3 and cleaved PARP in the A549-LIMD1 and H1299-LIMD1 groups were significantly higher than those in their respective control groups (A549-vec and H1299-vec). The aforementioned results showed that upon LIMD1 overexpression, lung adenocarcinoma cells had significantly increased levels of cisplatin-induced apoptosis compared to those of the control group, suggesting that LIMD1 overexpression increased the sensitivity of lung adenocarcinoma cells to cisplatin.

### LIMD1 Induces GADD45α Expression and Activates the p38 MAPK Signaling Pathway

**Figure 3:**
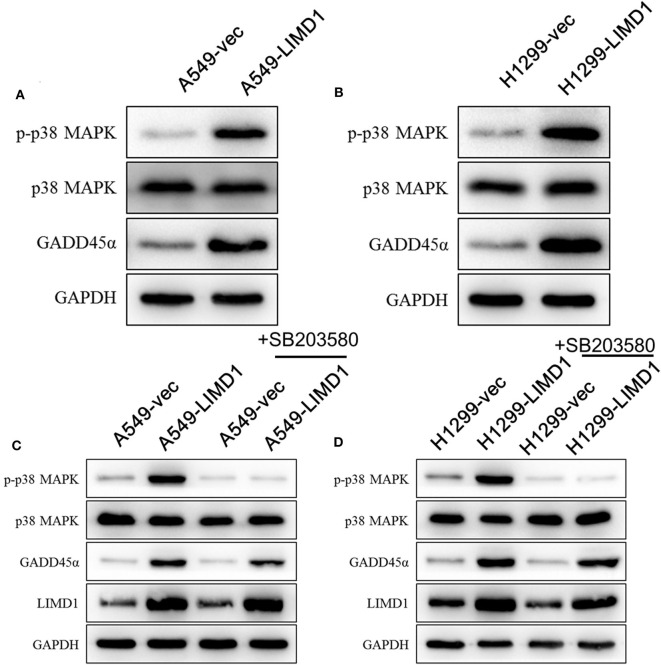
LIMD1 induces DNA damage-inducible 45 alpha (GADD45α) expression and activates the p38 mitogen-activated protein kinase (MAPK) signaling pathway. **(A)** Western blot analysis was used to detect the protein expression of GADD45α and constituents of the p38 MAPK signaling pathway in A549-vec and A549-LIMD1 cells. **(B)** Western blot analysis was used to detect the protein expression of GADD45α and constituents of the p38 MAPK signaling pathway in H1299-vec and H1299-LIMD1 cells. **(C)** The protein expression of constituents of the p38 MAPK signaling pathway, GADD45α and LIMD1, was detected by Western blot in A549-vec and A549-LIMD1 cells treated with SB203580; A549-vec and A549-LIMD1 cells treated with DMSO served as controls. **(D)** The protein expression of constituents of the p38 MAPK signaling pathway, GADD45α and LIMD1, was detected by Western blot in H1299-vec and H1299-LIMD1 cells treated with SB203580; H1299-vec and H1299-LIMD1 cells treated with DMSO served as controls.

To further elucidate the mechanism by which LIMD1 increases the sensitivity of lung adenocarcinoma cells to cisplatin, we detected the expression of GADD45α and constituents of the p38 MAPK signaling pathway. The levels of p-p38 MAPK in A549 cells overexpressing LIMD1 was significantly increased (Figure 3A), indicating that LIMD1 activates the p38 MAPK signaling pathway. We further detected the expression of GADD45α (upstream of the p38 MAPK pathway) and found that it was increased in LIMD1-overexpressing A549 cells, suggesting that LIMD1 activates the p38 MAPK pathway via GADD45α. A similar result was observed in H1299 cells. The levels of p-p38 MAPK and GADD45α were significantly increased in H1299 cells with LIMD1 overexpression (Figure 3B). The aforementioned results show that LIMD1 activates the p38 MAPK signaling pathway via GADD45α.

**Figure 4:**
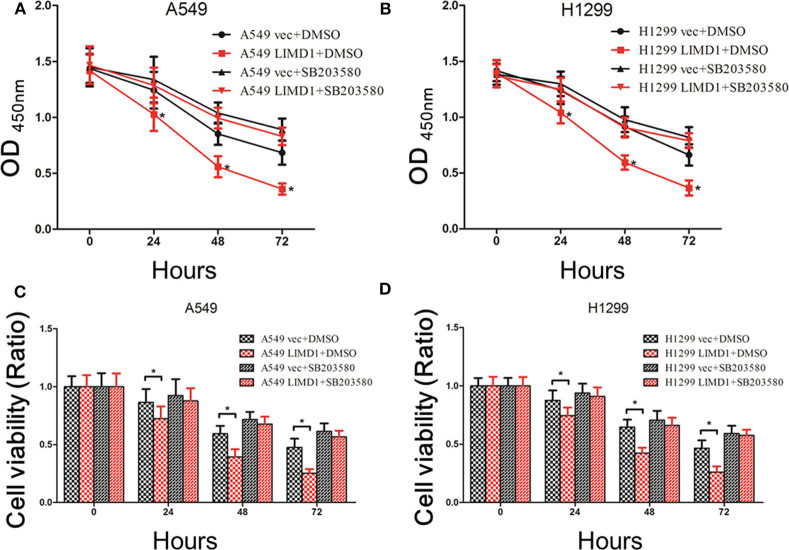
p38 MAPK inhibition abolished LIMD1-mediatedcell death. **(A)** After A549-vec and A549-LIMD1 cells were treated with cisplatin and DMSO or SB203580, respectively, the OD values were measured at different time points. **(B)** After H1299-vec and H1299-LIMD1 cells were treated with cisplatin and DMSO or SB203580, respectively, the OD values were measured at different time points. **(C)** After A549-vec and A549-LIMD1 cells were treated with cisplatin and DMSO or SB203580, respectively, the cell viability was calculated at different time points. **(D)** After H1299-vec, H1299-LIMD1 cells were treated with cisplatin and DMSO or SB203580, respectively, the cell viability was calculated at different time points. *A statistically significant difference at *P* < 0.05.

To further investigate whether the p38 MAPK signaling pathway is involved in the process of LIMD1 increasing the sensitivity of lung adenocarcinoma cells to cisplatin, we added 10-μM SB203580 (an inhibitor of the p38 MAPK signaling pathway) to A549-vec, A549-LIMD1, H1299-vec, and H1299-LIMD1 cells and determined the cellular protein expression by Western blot. As shown in Figure 4A, SB203580 significantly inhibited the activation of the p38 MAPK signaling pathway in A549-vec and A549-LIMD1 cells but had no significant effect on the expression of GADD45α or LIMD1 (Figure 3C). Similarly, SB203580 significantly inhibited the activation of the p38 MAPK signaling pathway in H1299-vec and H1299-LIMD1 cells but had no significant effect on the expression of GADD45α or LIMD1 (Figure 3D).

### LIMD1 Increases the Sensitivity of Lung Adenocarcinoma Cells to Cisplatin via the GADD45α/p38 MAPK Signaling Pathway

A549-vec, A549-LIMD1, H1299-vec, and H1299-LIMD1 cells were cotreated with cisplatin and either DMSO or SB203580, and cell proliferation and apoptosis were subsequently assessed. The CCK-8 results showed that the OD value and viability of A549-LIMD1 cells were significantly lower than those of A549-vec cells at 24, 48, and 72 h after treatment with cisplatin alone (*p* < 0.05) (Figures 4A,C), which is consistent with the abovementioned results. However, combined administration of SB203580 and cisplatin resulted in no significant difference in the OD value of between A549-vec and A549-LIMD1 cells at 24, 48, or 72 h (Figure 4A). Furthermore, the viability of A549-LIMD1 and A549-vec cells showed no significant difference after inhibiting the p38 MAPK signaling pathway (at 24, 48, 72 h after cisplatin treatment) (Figure 4C). Similar results were observed in H1299 cells: the OD value and viability of H1299-LIMD1 cells at 24, 48, and 72 h were significantly lower than those of H1299-vec cells in the absence of SB203580 (*p* < 0.05) (Figures 4B,D); however, upon cotreatment with SB203580 and cisplatin, there was no significant difference in the growth of H1299-vec and H1299-LIMD1 cells at 24, 48, and 72 h (Figure 4B). After inhibiting the p38 MAPK signaling pathway, there was no significant difference in cell viability between the two groups at 24, 48, and 72 h (**Figure 6**).

**Figure 5:**
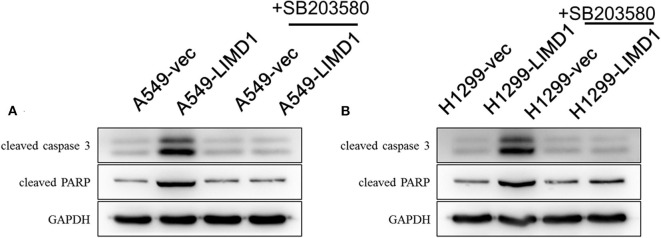
p38 MAPK inhibition abolished LIMD1-induced apoptosis in non-small-cell lung cancer. **(A)** A549-vec and A549-LIMD1 cells were cotreated with cisplatin and DMSO or SB203580, respectively, and the expression of proapoptotic proteins was detected. **(B)** H1299-vec and H1299-LIMD1 cells were cotreated with cisplatin and DMSO or cisplatin, respectively, and the expression of proapoptotic proteins was detected.

Subsequently, we further examined the expression of proapoptotic proteins in each group. DMSO or SB203580 was administered with 30-μM cisplatin to A549-vec, A549-LIMD1, H1299-vec, and H1299-LIMD1 cells, and the expression of proapoptotic proteins was detected by Western blot analysis. As shown in Figure 5A, the expression of proapoptotic proteins in the A549-LIMD1 group was significantly higher than that in the control group in response to the cisplatin + DMSO treatment, further supporting that LIMD1 overexpression increased the sensitivity of A549 cells to cisplatin. By contrast, administration of SB203580 inhibited the p38 MAPK pathway, so the expression of proapoptotic proteins in the A549-LIMD1 group was significantly decreased, i.e., inhibition of the p38 MAPK signaling pathway reversed the increase in the expression of apoptotic proteins induced by LIMD1 overexpression, suggesting that LIMD1 plays a regulatory role on cell proliferations via the p38 MAPK signaling pathway. Similar results were obtained in H1299-LIMD1 and H1299-vec cells (Figure 5B).

**Figure 6:**
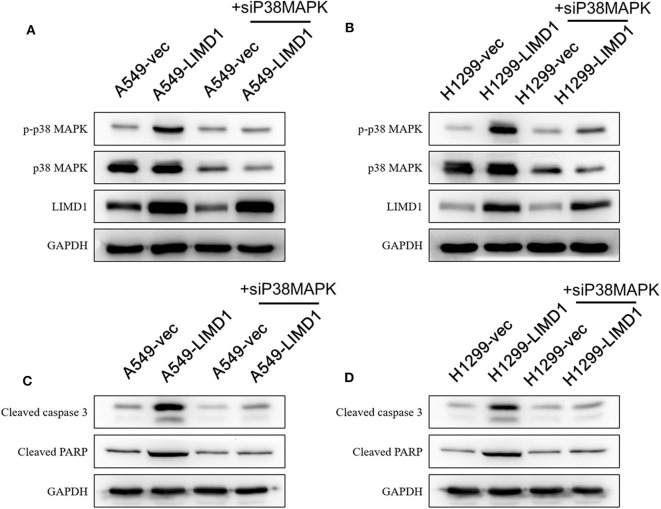
LIMD1 regulated the apoptotic effect of cisplatin on non-small-cell lung cancer via the p38 MAPK pathway. **(A,B)** A549-vec, A549-LIMD1, H1299-vec, and H1299-LIMD1 cells were transfected with p38 MAPK siRNA, and then the expression of proapoptotic proteins was detected by Western blot analysis. **(C,D)** A549-vec, A549-LIMD1, H1299-vec, and H1299-LIMD1 cells were transfected with p38 MAPK siRNA, and then the expression of proapoptotic proteins was detected by Western blot analysis at 24 h after cisplatin treatment.

In addition, A549-vec, A549-LIMD1, H1299-vec, and H1299-LIMD1 cells were transfected with p38 MAPK siRNA to knock down p38 MAPK gene expression and subsequently followed by cisplatin treatment. As shown in Figures 6A,B, in A549 and H1299 cells transfected with p38 MAPK siRNA, the expression of p38 MAPK was significantly decreased. The levels of p-p38 MAPK in A549-LIMD1 and H1299-LIMD1 cells were significantly decreased but presented no significant difference compared with the levels in the control groups. Interfering with p38 MAPK expression had no significant effect on LIMD1 expression. After treatment with cisplatin, A549 and H1299 cells transfected with p38 MAPK siRNA showed attenuation of LIMD1-induced apoptosis. In addition, in both cell lines, there was no significant difference in the expression of proapoptotic proteins between the control and LIMD1 overexpression groups after transfection with p38 MAPK siRNA.

These results confirmed that the p38 MAPK signaling pathway is involved in LIMD1-mediated increases in the sensitivity of lung adenocarcinoma cells to cisplatin. In summary, LIMD1 increases the sensitivity of lung adenocarcinoma cells to cisplatin by activating the GADD45α/p38 MAPK signaling pathway.

## Discussion

Recently, the incidence of lung adenocarcinoma has increased annually. Epidemiological data show that lung adenocarcinoma has become the main pathological type of lung cancer, with a higher incidence than that of lung squamous cell carcinoma (22). Chemotherapy is an important approach for treating lung adenocarcinoma, but most patients with lung adenocarcinoma gradually become less sensitive to chemotherapy, leading to treatment failure and poor prognosis (23). Therefore, increasing the sensitivity of lung adenocarcinoma in patients to chemotherapy drugs helps improve patient survival. LIMD1 plays a regulatory role in many biological functions in tumor cells (17, 20, 24, 25) and acts as a tumor suppressor (18). In this study, we investigated the effect of LIMD1 on the sensitivity of lung adenocarcinoma cells to chemotherapy drugs and explored the mechanism(s) involved.

In our study, LIMD1 overexpression in lung adenocarcinoma cells increased the sensitivity of lung adenocarcinoma cells to cisplatin, suggesting that LIMD1 is involved in regulating the susceptibility of lung cancer cells to this drug. The observed increases in the sensitivity of lung adenocarcinoma cells to cisplatin may be due to LIMD1 regulation through the GADD45α/p38 MAPK signaling pathway.

Recent studies have found that the decreased LIMD1 expression plays an important role in lung cancer progression; thus, LIMD1 may be an anticancer gene (16). In addition, LIMD1 is involved in the regulation of the cell cycle and mitosis (24, 26), and its low expression in tumors is associated with poor prognosis (27, 28). However, there are few studies on LIMD1-mediated regulation of drug sensitivity, and the mechanism involved remains to be elucidated. We detected cell viability with the CCK-8 assay and apoptosis by Western blotting and flow cytometry in lung cancer cells overexpressing LIMD1 and treated with cisplatin and found that the LIMD1 overexpression group had lower cell viability and higher rates of apoptosis than did the control group, suggesting that LIMD1 overexpression increases the sensitivity of lung cancer cells to cisplatin. Subsequently, we examined the expression of relevant proteins: the levels of p-p38 MAPK were increased in response to LIMD1 overexpression, suggesting that p38 MAPK signaling activity was increased; the expression of GADD45α, a protein upstream of the p38 MAPK signaling pathway, was also increased after LIMD1 overexpression. These results demonstrated that LIMD1 activates the p38 MAPK signaling pathway by upregulating GADD45α expression.

Previous studies have found that the p38 MAPK pathway is involved in the regulation of tumor apoptosis. 3,3′-Diindolylmethane (DIM), a compound isolated from cruciferous vegetables such as cabbage and broccoli, has antitumor effects. DIM can induce the apoptosis of liver cancer cells in a concentration-dependent manner by increasing the levels of p-p38 MAPK. DIM-induced apoptosis is significantly inhibited when cells are treated with SB203580, suggesting that the p38 MAPK signaling pathway is involved in DIM-induced apoptosis (29). This result is consistent with the findings of our study. We observed that cisplatin-induced expression of proapoptotic proteins was significantly reduced upon either inhibition of the p38 MAPK signaling pathway by SB203580 or knockdown of p38 MAPK protein expression by siRNA, suggesting that activation of the p38 MAPK pathway is related to the apoptotic process.

To further confirm whether the p38 MAPK signaling pathway is involved in the LIMD1-mediated increase in the sensitivity of lung adenocarcinoma cells to cisplatin, we either treated cells with SB203580 or transfected siRNA into cells to inhibit p38 MAPK signaling pathway activity. The results of the CCK-8 assay showed that upon SB203580-mediated inhibition of the p38 MAPK signaling pathway, there was no significant difference in the cell growth and viability between the LIMD1 overexpression group and the control group in the presence of cisplatin, indicating that LIMD1 could no longer increase the sensitivity of lung adenocarcinoma cells to cisplatin. Western blot analysis also showed that the expression of proapoptotic proteins in lung adenocarcinoma cells overexpressing LIMD1was significantly increased compared with that in the control cells. LIMD1 overexpression increased the sensitivity of lung adenocarcinoma cells to cisplatin, but after the p38 MAPK signaling pathway was inhibited by SB203580, the expression of proapoptotic proteins in the LIMD1-overexpressing group was significantly decreased, suggesting that inhibiting the p38 MAPK signaling pathway can reverse the increase in apoptotic protein expression induced by LIMD1 overexpression. Similarly, siRNA-mediated inhibition of p38 MAPK expression also reversed the increase in the expression of proapoptotic proteins in lung adenocarcinoma cells induced by LIMD1 overexpression. In conclusion, LIMD1 increases the sensitivity of lung adenocarcinoma cells to cisplatin via the GADD45α/p38 MAPK signaling pathway.

## Data Availability Statement

The original contributions presented in the study are included in the article/supplementary material, further inquiries can be directed to the corresponding author.

## Author Contributions

XZ performed the reseach and write the paper. RM designed the research. HW and DH performed the experiments and analyzed the data. WJ reviewed the whole paper. All authors contributed to the article and approved the submitted version.

### Conflict of Interest

The authors declare that the research was conducted in the absence of any commercial or financial relationships that could be construed as a potential conflict of interest.
